# *N*-(4-Methoxy­phen­yl)-*tert*-butane­sulfinamide

**DOI:** 10.1107/S1600536809042548

**Published:** 2009-10-23

**Authors:** Mrityunjoy Datta, Alan J. Buglass, Mark R. J. Elsegood

**Affiliations:** a Department of Chemistry, Korea Advanced Institute of Science and Technology, Daejeon 305-701, Republic of Korea; b Chemistry Department, Loughborough University, Loughborough LE11 3TU, England

## Abstract

In the title compound, C_11_H_17_NO_2_S, the mol­ecules inter­act head-to-tail through N—H⋯OS hydrogen bonds, giving discrete centrosymmetric cyclic dimers. The *N*—C_ar­yl_ bond length [1.4225 (14) Å] is inter­mediate between that in *N*-phenyl-*tert*-butane­sulfinamide [1.4083 (12) Å] and the *N*—C_alk­yl_ bond lengths in *N*-alkyl­alkanesulfinamides (1.470–1.530 Å), suggesting weaker delocalization of electrons over the N atom and the aromatic ring due to the presence of the 4-meth­oxy group.

## Related literature

For *N*-aryl­alkanesulfinamides, see: Datta *et al.* (2008 ▶, 2009 ▶). For *N*-alkyl­alkanesulfinamides, see: Sato *et al*. (1975 ▶); Schuckmann *et al.* (1978 ▶); Ferreira *et al.* (2005 ▶). For the synthesis, see: Stretter *et al.* (1969 ▶).
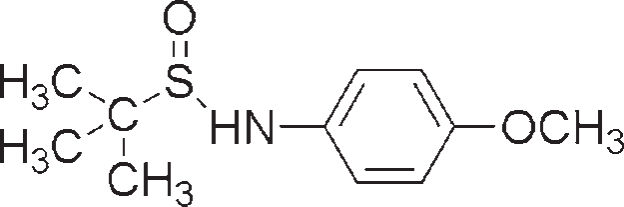


## Experimental

### 

#### Crystal data

C_11_H_17_NO_2_S
*M* *_r_* = 227.32
Orthorhombic, 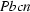

*a* = 19.6157 (11) Å
*b* = 9.1034 (5) Å
*c* = 13.3808 (7) Å
*V* = 2389.4 (2) Å^3^
*Z* = 8
Mo *K*α radiation
μ = 0.25 mm^−1^
*T* = 150 K
0.70 × 0.37 × 0.33 mm

#### Data collection

Bruker APEXII CCD-detector diffractometer
Absorption correction: multi-scan (**SADABS**; Sheldrick, 2007 ▶) *T*_min_ = 0.843, *T*_max_ = 0.921
26681 measured reflections
3659 independent reflections
3027 reflections with *I* > 2σ(*I*)
*R*_int_ = 0.033

#### Refinement

*R*[*F* ^2^ > 2σ(*F* ^2^)] = 0.039
*wR*(*F* ^2^) = 0.108
*S* = 1.05
3659 reflections
144 parameters
H atoms treated by a mixture of independent and constrained refinement
Δρ_max_ = 0.37 e Å^−3^
Δρ_min_ = −0.38 e Å^−3^

### 

Data collection: *APEX2* (Bruker, 2006 ▶); cell refinement: *SAINT* (Bruker, 2006 ▶); data reduction: *SAINT*; program(s) used to solve structure: *SHELXS97* (Sheldrick, 2008 ▶); program(s) used to refine structure: *SHELXL97* (Sheldrick, 2008 ▶); molecular graphics: *SHELXTL* (Sheldrick, 2008 ▶); software used to prepare material for publication: *SHELXTL* and local programs.

## Supplementary Material

Crystal structure: contains datablocks I, global. DOI: 10.1107/S1600536809042548/zs2015sup1.cif

Structure factors: contains datablocks I. DOI: 10.1107/S1600536809042548/zs2015Isup2.hkl

Additional supplementary materials:  crystallographic information; 3D view; checkCIF report

## Figures and Tables

**Table 1:** Hydrogen-bond geometry (Å, °)

*D*—H⋯*A*	*D*—H	H⋯*A*	*D*⋯*A*	*D*—H⋯*A*
N1—H1⋯O1^i^	0.867 (16)	2.062 (17)	2.9201 (14)	170.1 (14)

Symmetry code: (i) 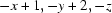.

**Fig. 1.**
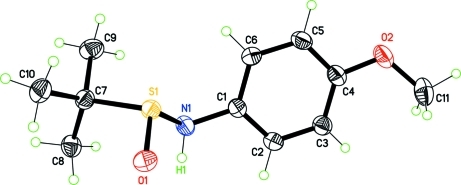
Molecular structure of (I), with atom labels and 50% probability displacement ellipsoids for non-H atoms.

**Fig. 2.**
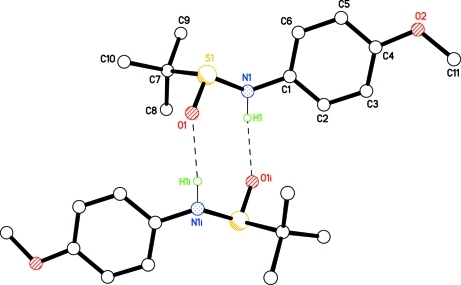
The centrosymmetric cyclic dimer of (I) in the crystal packing, showing intermolecular hydrogen bonding as dashed lines. Symmetry code: (i) -x + 1, -y + 2, -z.

## supplementary crystallographic information

### Comment

The molecular structure of (I) (Fig. 1) indicates a short *N*—C_aryl_ bond (1.4225 (14) Å), in contrast with *N*—C_alkyl_ bonds in *N*-alkylalkenesulfinamides (1.470–1.530 Å) (Sato *et al.*, 1975; Schuckmann *et al.*, 1978; Ferreira *et al.*, 2005). However, the *N*–C_aryl_ bond in (I) is longer than its equivalent in *N*-phenyladamantane-1-sulfinamide (1.409 (2) Å) (Datta *et al.*, 2008) and *N*-phenyl-*tert*-butanesulfinamide (1.4083 (12) Å) (Datta *et al.*, 2009), suggesting weaker delocalization of electrons over N and the aromatic ring due to the presence of the *para*-methoxy group. The crystal packing shows an intermolecular head-to-tail cyclic interaction through N—H···OS hydrogen bonds, forming discrete centrosymmetric dimers (Fig. 2 and Table 1). There is no evidence of any formal hydrogen bonding involving the methoxy group, nor of weak intermolecular C—H···OS hydrogen bonding, as observed in the packing of *N*-phenyladamantane-1-sulfinamide (Datta *et al.*, 2008).

### Experimental

Compound (I) was prepared by the method of Stretter *et al.* (1969), using *tert*-butanesulfinyl chloride (281 mg, 2 mmol) and 4-methoxyaniline (492 mg, 4 mmol) in dry diethyl ether (30 ml). After 5 h reaction time (with TLC monitoring), the white solid amine salt was filtered off and the solvent was removed under reduced pressure. Column chromatography (silica gel, 1% methanol-dichloromethane) yielded (I) as colourless crystals (420 mg (92%), m.p. 384 K. Single crystals suitable for X-ray analysis were obtained by evaporation of a solution of (I) in diethyl ether at room temperature. Spectroscopic analysis: FTIR (KBr) (cm^-1^) 3017, 1509, 1459, 1367, 1273, 1244, 1212, 1204, 1037, 866. ^1^H NMR (400 MHz, CDCl_3_, p.p.m. with respect to TMS) δ 6.96 (d, J = 8.8 Hz, 2H), 6.80 (d, J = 8.8 Hz, 2H), 5.20 (bs, 1H), 3.75 (s, 3H), 1.30 (s, 9H). ^13^C NMR (100 MHz, CDCl_3_, p.p.m. with respect to TMS) δ 156.2, 134.8, 121.5, 114.6, 56.2. 55.5, 22.4. EIMS m/*z* (%) 228 (MH^+^, 42), 227 (*M*^+^, 55), 122 (*M*^+^ - *^t^*BuSO, 100). These are the first recorded data for (I).

### Refinement

H atoms were located in a difference Fourier map and refined geometrically using a riding model except for N*H* for which the coordinates were freely refined. Bond lengths and displacement parameters were constrained as follows: C—H = 0.95–0.98 Å and with *U_iso_* (H) = 1.2 (1.5 for CH_3_) times *U_eq_*(C, N).

### Crystal data

**Table:**

C_11_H_17_NO_2_S	*D*_x_ = 1.264 Mg m^−^^3^
*M**_r_* = 227.32	Melting point: 384 K
Orthorhombic, *P**b**c**n*	Mo *K*α radiation, λ = 0.71073 Å
Hall symbol: -P 2n 2ab	Cell parameters from 6992 reflections
*a* = 19.6157 (11) Å	θ = 2.5–30.4°
*b* = 9.1034 (5) Å	µ = 0.25 mm^−^^1^
*c* = 13.3808 (7) Å	*T* = 150 K
*V* = 2389.4 (2) Å^3^	Block, colourless
*Z* = 8	0.70 × 0.37 × 0.33 mm
*F*(000) = 976	

### Data collection

**Table:**

Bruker APEXII CCD-detector diffractometer	3659 independent reflections
Radiation source: fine-focus sealed tube	3027 reflections with *I* > 2σ(*I*)
graphite	*R*_int_ = 0.033
ω rotation with narrow frames scans	θ_max_ = 30.6°, θ_min_ = 2.1°
Absorption correction: multi-scan (*SADABS*; Sheldrick, 2007)	*h* = −27→27
*T*_min_ = 0.843, *T*_max_ = 0.921	*k* = −13→12
26681 measured reflections	*l* = −18→19

### Refinement

**Table:**

Refinement on F^2^	Secondary atom site location: difference Fourier map
Least-squares matrix: full	H atoms treated by a mixture of independent and constrained refinement
R[F^2^ > 2σ(F^2^)] = 0.039	w = 1/[σ^2^(F_o_^2^) + (0.0614P)^2^ + 0.5032P] where P = (F_o_^2^ + 2F_c_^2^)/3
wR(F^2^) = 0.108	(Δ/σ)_max_ < 0.001
S = 1.05	Δρ_max_ = 0.37 e Å^−^^3^
3659 reflections	Δρ_min_ = −0.38 e Å^−^^3^
144 parameters	Extinction correction: SHELXL97>/i> (Sheldrick, 2008), Fc^*^=kFc[1+0.001xFc^2^λ^3^/sin(2θ)]^-1/4^
0 restraints	Extinction coefficient: 0.0049 (8)
Primary atom site location: structure-invariant direct methods	

### Special details

**Table:**

Geometry. All e.s.d.'s (except the e.s.d. in the dihedral angle between two l.s. planes) are estimated using the full covariance matrix. The cell e.s.d.'s are taken into account individually in the estimation of e.s.d.'s in distances, angles and torsion angles; correlations between e.s.d.'s in cell parameters are only used when they are defined by crystal symmetry. An approximate (isotropic) treatment of cell e.s.d.'s is used for estimating e.s.d.'s involving l.s. planes.
Refinement. Refinement of *F*^2^ against ALL reflections. The weighted *R*-factor *wR* and goodness of fit *S* are based on *F*^2^, conventional *R*-factors *R* are based on *F*, with *F* set to zero for negative *F*^2^. The threshold expression of *F*^2^ > σ(*F*^2^) is used only for calculating *R*-factors(gt) *etc*. and is not relevant to the choice of reflections for refinement. *R*-factors based on *F*^2^ are statistically about twice as large as those based on *F*, and *R*- factors based on ALL data will be even larger.

### Fractional atomic coordinates and isotropic or equivalent isotropic displacement parameters (Å^2^)

**Table:**

	*x*	*y*	*z*	*U*_iso_*/*U*_eq_	
O1	0.55068 (4)	1.13801 (9)	0.06229 (6)	0.02755 (19)	
S1	0.613894 (14)	1.06197 (3)	0.02673 (2)	0.02307 (10)	
N1	0.59358 (6)	0.89272 (11)	−0.01268 (8)	0.0256 (2)	
H1	0.5522 (8)	0.8854 (16)	−0.0349 (11)	0.031*	
C1	0.61179 (5)	0.77090 (13)	0.04831 (8)	0.0229 (2)	
C2	0.56489 (6)	0.65860 (12)	0.06430 (9)	0.0258 (2)	
H2	0.5208	0.6652	0.0353	0.031*	
C3	0.58165 (6)	0.53688 (13)	0.12200 (9)	0.0281 (2)	
H3	0.5491	0.4613	0.1328	0.034*	
C4	0.64632 (6)	0.52643 (13)	0.16383 (9)	0.0283 (2)	
C5	0.69388 (6)	0.63664 (14)	0.14644 (9)	0.0302 (3)	
H5	0.7383	0.6288	0.1743	0.036*	
C6	0.67709 (6)	0.75779 (13)	0.08881 (9)	0.0275 (2)	
H6	0.7101	0.8321	0.0768	0.033*	
O2	0.66770 (5)	0.41138 (10)	0.22206 (8)	0.0405 (2)	
C11	0.61629 (8)	0.32282 (15)	0.26749 (11)	0.0421 (3)	
H11A	0.5832	0.3862	0.3014	0.063*	
H11B	0.6374	0.2565	0.3162	0.063*	
H11C	0.5930	0.2650	0.2160	0.063*	
C7	0.63507 (6)	1.14369 (12)	−0.09505 (8)	0.0251 (2)	
C8	0.57849 (6)	1.11814 (14)	−0.17123 (9)	0.0296 (2)	
H8A	0.5877	1.1761	−0.2314	0.044*	
H8B	0.5347	1.1482	−0.1425	0.044*	
H8C	0.5767	1.0137	−0.1887	0.044*	
C9	0.70236 (7)	1.07343 (15)	−0.12670 (11)	0.0351 (3)	
H9A	0.6963	0.9670	−0.1330	0.053*	
H9B	0.7373	1.0941	−0.0763	0.053*	
H9C	0.7167	1.1142	−0.1912	0.053*	
C10	0.64515 (7)	1.30746 (13)	−0.07418 (10)	0.0321 (3)	
H10A	0.6645	1.3551	−0.1334	0.048*	
H10B	0.6763	1.3198	−0.0175	0.048*	
H10C	0.6011	1.3525	−0.0582	0.048*	

### Atomic displacement parameters (Å^2^)

**Table:**

	*U*^11^	*U*^22^	*U*^33^	*U*^12^	*U*^13^	*U*^23^
O1	0.0260 (4)	0.0291 (4)	0.0275 (4)	0.0000 (3)	0.0027 (3)	−0.0045 (3)
S1	0.02289 (15)	0.02413 (15)	0.02218 (15)	−0.00033 (9)	−0.00198 (9)	−0.00084 (9)
N1	0.0277 (5)	0.0220 (4)	0.0272 (5)	0.0001 (4)	−0.0057 (4)	0.0009 (4)
C1	0.0246 (5)	0.0237 (5)	0.0205 (5)	0.0034 (4)	0.0007 (4)	−0.0007 (4)
C2	0.0238 (5)	0.0256 (5)	0.0279 (5)	0.0019 (4)	−0.0032 (4)	−0.0012 (4)
C3	0.0282 (6)	0.0250 (5)	0.0310 (6)	−0.0009 (4)	−0.0030 (4)	0.0013 (4)
C4	0.0305 (6)	0.0272 (5)	0.0272 (5)	0.0059 (5)	−0.0021 (4)	0.0017 (4)
C5	0.0229 (5)	0.0366 (6)	0.0310 (6)	0.0059 (5)	−0.0022 (4)	0.0030 (5)
C6	0.0220 (5)	0.0309 (6)	0.0295 (6)	0.0003 (4)	0.0022 (4)	0.0028 (4)
O2	0.0408 (5)	0.0357 (5)	0.0451 (6)	0.0047 (4)	−0.0105 (4)	0.0147 (4)
C11	0.0590 (9)	0.0309 (6)	0.0363 (7)	−0.0034 (6)	−0.0074 (6)	0.0087 (5)
C7	0.0251 (5)	0.0245 (5)	0.0256 (5)	−0.0002 (4)	0.0021 (4)	0.0009 (4)
C8	0.0336 (6)	0.0314 (6)	0.0238 (5)	−0.0004 (5)	−0.0017 (4)	0.0017 (4)
C9	0.0274 (6)	0.0388 (7)	0.0392 (7)	0.0021 (5)	0.0070 (5)	−0.0028 (5)
C10	0.0339 (6)	0.0266 (5)	0.0358 (6)	−0.0045 (5)	0.0021 (5)	0.0007 (5)

### Geometric parameters (Å, °)

**Table:**

O1—S1	1.4977 (9)	O2—C11	1.4271 (18)
S1—N1	1.6765 (10)	C11—H11A	0.9800
S1—C7	1.8388 (11)	C11—H11B	0.9800
N1—C1	1.4225 (14)	C11—H11C	0.9800
N1—H1	0.867 (16)	C7—C8	1.5246 (16)
C1—C2	1.3919 (16)	C7—C9	1.5267 (17)
C1—C6	1.3960 (16)	C7—C10	1.5296 (16)
C2—C3	1.3900 (16)	C8—H8A	0.9800
C2—H2	0.9500	C8—H8B	0.9800
C3—C4	1.3897 (17)	C8—H8C	0.9800
C3—H3	0.9500	C9—H9A	0.9800
C4—O2	1.3711 (14)	C9—H9B	0.9800
C4—C5	1.3896 (18)	C9—H9C	0.9800
C5—C6	1.3854 (16)	C10—H10A	0.9800
C5—H5	0.9500	C10—H10B	0.9800
C6—H6	0.9500	C10—H10C	0.9800
			
O1—S1—N1	109.15 (5)	O2—C11—H11C	109.5
O1—S1—C7	106.35 (5)	H11A—C11—H11C	109.5
N1—S1—C7	98.44 (5)	H11B—C11—H11C	109.5
C1—N1—S1	118.44 (8)	C8—C7—C9	112.33 (10)
C1—N1—H1	111.8 (10)	C8—C7—C10	111.40 (10)
S1—N1—H1	113.7 (10)	C9—C7—C10	110.31 (10)
C2—C1—C6	118.95 (11)	C8—C7—S1	111.49 (8)
C2—C1—N1	119.66 (10)	C9—C7—S1	105.76 (8)
C6—C1—N1	121.32 (11)	C10—C7—S1	105.17 (8)
C3—C2—C1	120.96 (11)	C7—C8—H8A	109.5
C3—C2—H2	119.5	C7—C8—H8B	109.5
C1—C2—H2	119.5	H8A—C8—H8B	109.5
C4—C3—C2	119.62 (11)	C7—C8—H8C	109.5
C4—C3—H3	120.2	H8A—C8—H8C	109.5
C2—C3—H3	120.2	H8B—C8—H8C	109.5
O2—C4—C5	116.18 (11)	C7—C9—H9A	109.5
O2—C4—C3	124.09 (11)	C7—C9—H9B	109.5
C5—C4—C3	119.73 (11)	H9A—C9—H9B	109.5
C6—C5—C4	120.56 (11)	C7—C9—H9C	109.5
C6—C5—H5	119.7	H9A—C9—H9C	109.5
C4—C5—H5	119.7	H9B—C9—H9C	109.5
C5—C6—C1	120.14 (11)	C7—C10—H10A	109.5
C5—C6—H6	119.9	C7—C10—H10B	109.5
C1—C6—H6	119.9	H10A—C10—H10B	109.5
C4—O2—C11	117.22 (11)	C7—C10—H10C	109.5
O2—C11—H11A	109.5	H10A—C10—H10C	109.5
O2—C11—H11B	109.5	H10B—C10—H10C	109.5
H11A—C11—H11B	109.5		
			
O1—S1—N1—C1	−106.05 (9)	C4—C5—C6—C1	0.60 (19)
C7—S1—N1—C1	143.29 (9)	C2—C1—C6—C5	−1.93 (17)
S1—N1—C1—C2	135.83 (10)	N1—C1—C6—C5	−178.79 (11)
S1—N1—C1—C6	−47.33 (14)	C5—C4—O2—C11	160.40 (12)
C6—C1—C2—C3	1.88 (17)	C3—C4—O2—C11	−20.22 (18)
N1—C1—C2—C3	178.79 (11)	O1—S1—C7—C8	−61.74 (9)
C1—C2—C3—C4	−0.47 (18)	N1—S1—C7—C8	51.16 (9)
C2—C3—C4—O2	179.75 (12)	O1—S1—C7—C9	175.89 (8)
C2—C3—C4—C5	−0.89 (18)	N1—S1—C7—C9	−71.21 (9)
O2—C4—C5—C6	−179.76 (11)	O1—S1—C7—C10	59.13 (9)
C3—C4—C5—C6	0.83 (19)	N1—S1—C7—C10	172.03 (8)

### Hydrogen-bond geometry (Å, °)

**Table:**

*D*—H···*A*	*D*—H	H···*A*	*D*···*A*	*D*—H···*A*
N1—H1···O1^i^	0.867 (16)	2.062 (17)	2.9201 (14)	170.1 (14)

Symmetry codes: (i) −*x*+1, −*y*+2, −*z*.
